# Supplementary material for: Evaluating the Properties of Ginger Protease-Degraded Collagen Hydrolysate and Identifying the Cleavage Site of Ginger Protease by Using an Integrated Strategy and LC-MS Technology
Source: Molecules. 2022 Aug 6;27(15):5001. doi: 10.3390/molecules27155001 (PMC9370692; doi:10.3390/molecules27155001)
Supplement: Supplementary file 1 [file molecules-27-05001-s001.zip › molecules-1825374-supplementary.pdf]

Table S1 peptides from the GDCH identified by nano LC-MS/MS

No.	Peptide sequence	Mass/Da	Length	m/z	z	t_R	PTM
1	KAPDPFR	829.4446	7	277.4877	3	14.07	
2	FSGLDGAK	793.397	8	397.7046	2	17.63	
3	GERGPXGPM	826.4185	9	827.4235	1	10.45	
4	GFSGLDGAK	850.4185	9	426.2151	2	19.01	
5	GPDGYSGPV	799.3485	9	800.3513	1	11.51	Pro->Hyp
6	GPXGTMGPA	1005.4578	9	503.7372	2	10	Pro->Hyp
7	GPSGFXGPK	847.3712	9	848.373	1	23.71	Deamidation (NQ)
8	GDRGFXGER	858.4186	9	859.428	1	13.97	Pro->Hyp
9	GRNGEMGPA	842.4246	9	843.4268	1	8.57	
10	GPDGKIGPS	857.4233	9	858.4288	1	41.91	Pro->Hyp
11	XGIGFPGPT	911.4775	9	912.4863	1	8.69	Pro->Hyp
12	KXGDRGIPG	912.4074	9	457.2124	2	12.18	Pro->Hyp
13	FXGERGAXG	918.4097	9	919.4283	1	11.35	Pro->Hyp
14	AEGRTGPVG	887.3919	9	888.3942	1	9.33	
15	GPXGPXGPGP	984.4988	10	985.5076	1	7.92	
16	GPPVPGPIGP	886.4912	10	887.4932	1	34.97	
17	GXPGERGFXG	938.4933	10	939.499	1	7.48	
18	GPXGPPGPGP	1001.4468	10	1002.4722	1	13.17	Pro->Hyp; Deamidation (NQ)
19	GPVGKDGRPG	828.413	10	829.4169	1	18.7	
20	GPPGPPGPGP	844.403	10	845.4099	1	16.47	Pro->Hyp
21	GFTGPXGEXG	1001.4468	10	501.74	2	13.19	Pro->Hyp; Deamidation (NQ)
22	GPXGERGFXG	860.3929	10	861.4067	1	13.71	Pro->Hyp
23	GLDGRKGEPG	946.3934	10	947.4068	1	18.65	Pro->Hyp

24	KDGVDGIXGPI	1145.5449	11	1146.5535	1	7.85	Pro->Hyp
25	QDGRXGPXGPG	1000.4825	11	1001.4888	1	9.46	
26	NAGPXGPXGPA	1037.5142	11	1038.5189	1	14.08	
27	QDGRPGPXGPG	1085.5416	11	1086.5507	1	6.77	Pro->Hyp
28	KDGVDGIPGPI	952.4614	11	953.4655	1	8.67	
29	GRGFXGSDGPA	1053.5042	11	1054.515	1	12.03	Pro->Hyp
30	NPGPDGKIGPS	1082.5558	11	1083.5641	1	30.53	Deamidation (NQ); Pro->Hyp
31	KXGERGTMGPT	1031.4769	11	1032.4844	1	11.94	Pro->Hyp
32	SGPPVPGPIGP	1137.5792	11	1138.5925	1	8.47	Pro->Hyp
33	PGXGGPXGPXG	1066.5658	11	1067.5687	1	35.04	Deamidation (NQ)
34	ERGEAGPXGPA	1152.5901	11	577.3057	2	6.15	Pro->Hyp
35	XAGIVGPXGPA	962.4359	11	963.4503	1	13.13	Pro->Hyp
36	NXGPDGKIGPS	968.4514	11	969.4614	1	7.86	Pro->Hyp
37	KXGDRGAXGPQ	963.4927	11	964.5106	1	23.43	Pro->Hyp
38	GGSRGQXGIMG	1032.4575	11	517.2372	2	15.53	Pro->Hyp
39	KXGERGATGPT	1052.4838	11	1053.4935	1	9.07	Pro->Hyp
40	SXGPDGKAGPA	973.5233	11	974.527	1	35.79	
41	LDGAKGDTGPA	989.5496	11	990.5625	1	34.13	Pro->Hyp
42	SPGPDGKAGPA	1065.474	11	1066.4877	1	8.55	Pro->Hyp
43	LXGERGRXGPA	1049.484	11	1050.4921	1	10.13	Pro->Hyp
44	LPGIVGPXGPA	933.4044	11	934.4227	1	10.87	Pro->Hyp
45	KXGRXGERGPA	1110.5319	11	556.2765	2	6.73	Pro->Hyp
46	GNAGPXGPXGPA	1057.5039	12	1058.5081	1	11.99	
47	GHRGFSGLDGAK	1043.4883	12	1044.4932	1	10.77	
48	GLDGAKGDTGPA	1144.5472	12	1145.551	1	7.02	

49	GQDGRSGPXGPS	1200.6	12	301.1561	4	11.66	
50	GLDGAKGDSGPA	1126.4954	12	1127.5055	1	7.61	Pro->Hyp
51	GPXGLXGPXGPA	1094.5356	12	1095.5409	1	14.11	
52	GNPGPDGKIGPS	1033.473	12	1034.488	1	13.27	Pro->Hyp
53	GPXGIVGPXGPA	1111.4845	12	1112.4918	1	8.54	Pro->Hyp
54	GPXGAQGPXGAP	1019.4573	12	1020.4724	1	12.87	Pro->Hyp
55	GKTGDRGETGPA	1110.5206	12	1111.537	1	8.28	Pro->Hyp
56	GPAGRVGPXGPA	1060.5042	12	1061.5231	1	24.47	Pro->Hyp
57	GPXGLXGPPGPA	1044.5142	12	1045.5305	1	28.12	Pro->Hyp
58	GSXGDRGEPGPA	1047.5411	12	524.7792	2	13.13	Pro->Hyp
59	GPXGLXGPPGTP	1111.5258	12	1112.5287	1	10.42	
60	GPSGERGEVGPA	1074.5247	12	1075.5383	1	28.56	Pro->Hyp
61	GPXGVDGQXGAK	1046.5298	12	1047.5446	1	26.15	Pro->Hyp
62	XGPXGEEGKRGAR	1225.6001	13	1226.6061	1	9.34	Pro->Hyp
63	GDRGFXGERGAXG	1289.5649	13	645.7906	2	10.23	Pro->Hyp
64	GPSGPXGPXGPXG	1241.5901	13	1242.599	1	7.96	Pro->Hyp
65	GPHGPVGKDGRPG	1229.6265	13	410.8821	3	7.6	
66	GARGAXGSRGMXG	1273.6035	13	637.8088	2	8.02	Pro->Hyp
67	GDRGFXGERGGXG	1158.4843	13	1159.4969	1	19.3	Pro->Hyp
68	GFAGPXGADGQXG	1245.6165	13	623.817	2	7.07	Pro->Hyp
69	GPXGPXGEKGSPG	1124.457	13	1125.4664	1	8.22	Pro->Hyp
70	AGKXGERGTMGPT	1181.5577	13	1182.5698	1	9.47	Pro->Hyp
71	GEAGKXGDRGIPG	1164.5312	13	1165.5427	1	8.48	Pro->Hyp
72	GEAGKXGDRGIXG	1139.527	13	1140.5382	1	8.18	Pro->Hyp
73	GPHGPVGKDGRXG	1117.4891	13	1118.5088	1	12.46	Pro->Hyp

74	AGPXGVDGQXGAK	1201.5524	13	401.5269	3	7.44	Pro->Hyp
75	AGPXGADGQAGAR	1303.5807	13	652.8009	2	11.14	Pro->Hyp
76	GAXGHQGAGGMXG	1233.4912	13	617.7544	2	6.42	Pro->Hyp
77	GHTGHAGEXGEXG	1338.6542	13	447.2264	3	7.24	Pro->Hyp
78	GKGAXGERGAXGPL	1291.6044	14	1292.6056	1	24.32	
79	HKGPDGNXGRDGPR	1277.5887	14	1278.592	1	22.08	
80	AXGQDGRXGPXGPG	1221.5576	14	1222.5624	1	10.07	Pro->Hyp
81	FSGLDGAKGDTGPA	1254.5427	14	1255.5493	1	8.77	Pro->Hyp
82	ARGLXGERGRXGAP	1294.6531	14	1295.6652	1	10.94	Pro->Hyp
83	SRGEXGPDGAVGPV	1237.5476	14	1238.5609	1	8.5	Pro->Hyp
84	FSGLDGAKGDSGPA	1474.6975	14	738.3546	2	6.32	Pro->Hyp
85	ARGLXGERGRPGAP	1306.5754	14	654.2995	2	9	Pro->Hyp
86	XAGNAGPXGPXGPA	1436.6973	14	719.3602	2	6.44	Pro->Hyp
87	AXGPDGGKGEPGPA	1476.7285	14	739.3734	2	7.07	Pro->Hyp
88	AGPXGPXGEKGSPG	1235.5684	14	1236.5797	1	9.21	Pro->Hyp
89	AXGPDGGKGEXGPA	1309.6213	14	655.8179	2	18.95	Pro->Hyp; Deamidation (NQ)
90	RXGKXGDRGAXGPQ	1405.7489	14	469.5902	3	10.04	Pro->Hyp
91	DNGAKGDSGPXGPA	1203.5372	14	1204.5564	1	13.37	Pro->Hyp
92	KXGRXGERGPXGPQ	1421.7389	14	711.8775	2	8.56	Pro->Hyp
93	GAXGQDGRXGPXGPG	1348.6259	15	1349.6255	1	25.9	
94	GPXGERGEAGPXGPA	1459.6676	15	730.8386	2	8.47	Pro->Hyp
95	GSRGEXGPDGAVGPV	1260.5587	15	1261.5752	1	14.02	Pro->Hyp
96	GAXGQDGRPGPXGPG	1376.6222	15	689.3193	2	11.15	Pro->Hyp
97	GAXGQDGRXGPPGPG	1319.5741	15	1320.5708	1	15.77	
98	GFSGLDGAKGDSGPA	1306.6055	15	1307.6107	1	9.94	Pro->Hyp

99	GHKGPDGNXGRDGPR	1366.6427	15	1367.6514	1	18.63	Pro->Hyp; Deamidation (NQ)
100	GPXGPXGEEGKRGAR	1334.6102	15	668.308	2	26.28	
101	GFSGLDGAKGDTGPA	1363.5968	15	1364.6129	1	9.84	Pro->Hyp
102	GAXGPDGGKGEPGPA	1347.6068	15	674.8125	2	11.51	Pro->Hyp
103	AAGPXGPXGEKGSPG	1294.5691	15	1295.5796	1	8.76	Pro->Hyp
104	GKGAXGERGAXGPLG	1351.6746	15	676.8466	2	10.71	Pro->Hyp
105	GXAGNAGPXGPXGPA	1531.719	15	383.9369	4	6.4	Pro->Hyp
106	GAXGQDGRPGPPGPG	1295.5531	15	1296.5658	1	14.74	Pro->Hyp
107	GARGLXGERGRPGAP	1476.7384	15	370.1919	4	7.55	Pro->Hyp
108	GPSGQDGRSGPXGPS	1462.7703	15	488.5971	3	10.29	Pro->Hyp
109	GPPGPXGEEGKRGAR	1385.6486	15	693.8289	2	7.28	Pro->Hyp
110	GAAGRXGNRGEAGPS	1278.5791	15	1279.582	1	10.52	Pro->Hyp
111	GARGLXGERGRXGPA	1478.7604	15	740.3877	2	8.73	Pro->Hyp
112	GEAGKXGERGTMGPT	1367.6016	15	684.8073	2	8.18	Pro->Hyp
113	G(+42.01)PAGNAGPXGPXGPA	1331.6168	15	666.8146	2	12.74	Pro->Hyp
114	GPXGPXGTSGESGPA	1347.6068	15	674.8116	2	10.99	Pro->Hyp
115	GAXGPDGGKGEXGPA	1368.6444	15	685.3278	2	6.91	Pro->Hyp
116	GXAGKTGDRGETGPA	1492.7284	15	747.3734	2	6.91	Pro->Hyp
117	GANGDKGEGGSFGPA	1286.5792	15	1287.5939	1	14.46	Acetylation (N-term); Pro->Hyp
118	GGXGERGAXGGRGFXG	1587.7404	16	530.2543	3	9.49	Pro->Hyp
119	GARGDRGFXGERGAXG	1540.6969	16	771.3582	2	9.91	Pro->Hyp
120	GEAGKXGDRGIXGEXG	1472.6609	16	737.34	2	10.01	Pro->Hyp
121	GVGPSGKTGDRGESGPS	1601.7418	17	801.8762	2	9.11	Pro->Hyp
122	AAGEAGKXGERGTMGPT	1543.7227	17	772.8666	2	8.28	

123	GHRGFSGLDGAKGDTGPA	1698.8074	18	567.275	3	16.97	
124	ARGGXGERGAXGGRGFXG	1658.7633	18	830.3845	2	9.59	Pro->Hyp
125	GHRGFSGLDGAKGDSGPA	1604.7031	18	803.361	2	10.3	Pro->Hyp
126	GGAGGKGAXGERGAXGPL	1536.7545	18	769.3842	2	11.76	Pro->Hyp
127	GPSGAXGQDGRXGPXGPG	1699.7991	18	567.6098	3	8.69	Pro->Hyp
128	GAAGEAGKXGERGTMGPT	1684.7916	18	562.6024	3	15.9	
129	GARGGXGERGAXGGRGFXG	1848.8566	19	925.4402	2	7.48	Pro->Hyp
130	GPXGPXGEEGKRGARGEXG	1756.8206	19	586.6171	3	8.66	Pro->Hyp
131	ADGGAGGKGAXGERGAXGPL	1722.8186	20	862.4113	2	13.2	Pro->Hyp
132	REGTXGNEGAAGRDGAXGPK	1924.8888	20	642.6371	3	7.32	Pro->Hyp
133	ADGGAGGKGAXGERGAXGPLG	1779.8401	21	594.2888	3	12.68	Pro->Hyp
134	KAGEDGNNGRXGKXGDRGAXGPQ	2279.0491	23	760.6912	3	6.76	Pro->Hyp
135	GFXGADGGAGGKGAXGERGAXGPL	2096.9727	24	1049.4952	2	24.47	Pro->Hyp
136	ARGFXGADGGAGGKGAXGERGAX GPL	2324.1108	26	582.0366	4	21.11	Pro->Hyp

X:hydroxyproline.

Table S2 The analysis certificate of gelatin supplied by Shanghai Xinxi Biotechnology Co.

Test	Specification	Test result	Test Method
**Physicochemical parameter**			
Transparency (10%solution)	≤25 NTU	≤21.31 NTU	Ref.TCVN 6184:2008
Appearance	Yellow to amber color powder	Yellow powder	Visual
Foreign material	Not detected	Not detected	Mental detector
pH value of 1% solution	5.0-7.0	5.42	USP
Gel strength (Bloom) 6.67%	250±15	243.1 g	USP
Viscosity (6.67%, 60℃)	≥ 30 mps<br>(≥ 3.0 mPa.s)	35.6 mps<br>(3.56 mPa.s)	GMIA
Moisture content	≤12%	9.31%	USP
(Residue on ignition)<br>Total ash content	≤2%	0.27%	AOAC
Protein content	≥ 85%	89.2%	FAO
Particle size	≥ 95% through 8 mesh size	95.37% through 8 mesh	
Sulfit*	<3mg/kg	Pass	AOAC
Melamine	<2.5 ppm	Not detected	QQQ-LC/MS/MS-Ref FDA (USA)
**Heavy metal**			
Pb*	≤ 1.0 ppm	Pass	AOAC
As*	≤ 1.0 ppm	Pass	AOAC
Cr*	≤ 10 ppm	Pass	AOAC
**Microbiological parameter**			

Total Plate Count	≤ 1000 cfu/g	<10 cfu/g	USP
Total yeast and mould	<100 cfu/g	<10 cfu/g	USP
Escherichia Coli	Not detected	Not detected	USP
Coliforms	Not detected	Not detected	ISO 4831:2006
Salmonella	Negative/25g	Negative/25g	USP
**Hydroxyproline content**			
**7.258±0.089%**[&]			

[*]Performed on routine level, as per quality control plan.

[&] It was conducted using the alkali hydrolysis method with a commercially available kit (Jiangsu Kaiji Biotechnology Co. Nanjing, China)
